# DNA Barcoding as an Effective Tool in Improving a Digital Plant Identification System: A Case Study for the Area of Mt. Valerio, Trieste (NE Italy)

**DOI:** 10.1371/journal.pone.0043256

**Published:** 2012-09-10

**Authors:** Ilaria Bruni, Fabrizio De Mattia, Stefano Martellos, Andrea Galimberti, Paolo Savadori, Maurizio Casiraghi, Pier Luigi Nimis, Massimo Labra

**Affiliations:** 1 Università degli Studi di Milano-Bicocca, ZooPlantLab, Dipartimento di Biotecnologie e Bioscienze, Piazza della Scienza 2, Milano, Italy; 2 Università degli Studi di Trieste, Dipartimento di Scienze della Vita, via L. Giorgieri 10, Trieste, Italy; University of Alberta, Canada

## Abstract

**Background:**

Identification keys are decision trees which require the observation of one or more morphological characters of an organism at each step of the process. While modern digital keys can overcome several constraints of classical paper-printed keys, their performance is not error-free. Moreover, identification cannot be always achieved when a specimen lacks some morphological features (i.e. because of season, incomplete development or miss-collecting). DNA barcoding was proven to have great potential in plant identification, while it can be ineffective with some closely related taxa, in which the relatively brief evolutionary distance did not produce differences in the core-barcode sequences.

**Methodology/Principal Findings:**

In this paper, we investigated how the DNA barcoding can support the modern digital approaches to the identification of organisms, using as a case study a local flora, that of Mt. Valerio, a small hill near the centre of Trieste (NE Italy). The core barcode markers (plastidial *rbcL* and *matK*), plus the additional *trnH-psbA* region, were used to identify vascular plants specimens. The usefulness of DNA barcoding data in enhancing the performance of a digital identification key was tested on three independent simulated scenarios.

**Conclusions/Significance:**

Our results show that the core barcode markers univocally identify most species of our local flora (96%). The *trnH-psbA* data improve the discriminating power of DNA barcoding among closely related plant taxa. In the multiparametric digital key, DNA barcoding data improves the identification success rate; in our simulation, DNA data overcame the absence of some morphological features, reaching a correct identification for 100% of the species. FRIDA, the software used to generate the digital key, has the potential to combine different data sources: we propose to use this feature to include molecular data as well, creating an integrated identification system for plant biodiversity surveys.

## Introduction

In biology, the identification process consists of assigning an existing taxon name to a specimen. Although related to classification, which is the job of taxonomists, identification belongs to a different operational process [1]. Correct identification can be a necessity for physiologists, pharmacologists, conservation biologists, technical personnel of environmental agencies, or just fun for laypersons [2]. Ideally, an identification tool should allow users to reach a positive identification irrespective of their level of expertise. Reality is, however, quite different. Until a few years ago, identification was mostly based on dichotomous or polytomous keys printed on paper. The constraints of a paper-printed publication forced most authors to organize data according to the hierarchical scheme of biological classification, so that most classical identification keys first lead to families, then to genera, and finally to species [3]. However, diagnostic characters of higher taxonomic ranks are usually difficult to understand and observe, even for users with average skills, which makes “classic” keys intrinsically difficult. Furthermore, paper-printed keys are made of a series of identification steps fixed by the author(s), which must be followed entirely to obtain a correct identification. In the case of plants, this process could be time consuming even for skilled botanists. Moreover, immature or ruined specimens can be impossible to identify due to the lack of one or more fundamental characters. Digital identification keys can overcome these drawbacks. When they are based upon matrices of taxa and characters, they prevent users from following a fixed sequence of identification steps, and/or difficult characters. Digital keys can also include ecological and biogeographical characters, which are normally alien to the systematic scheme of classical keys. There exist several software packages for the creation of digital keys [4]–[8]. FRIDA (FRiendly IdentificAtion, [9]), which was developed at the Department of Life Sciences of the University of Trieste, produces keys which can be published on the Web, stored on optical devices, and used both online and as stand-alone packages on Smartphones and Tablets.

During the last decades, several molecular approaches to the identification of organisms have been explored [10]. Hebert and colleagues, pioneering the idea of a universal DNA barcoding system, used DNA sequence data from standard genome regions to identify organisms [11].

DNA barcoding is based on three keystones of modern taxonomy: molecularization (i.e. the use of the variability of molecular markers as discriminators; [12]); computerization (i.e. the non-redundant transposition of the data using informatics; [13]); and standardization (i.e. the extension of an approach to wide groups of not strictly related organisms). DNA barcoding was proven to perform well on metazoans [11], by using the mitochondrial *cox1* (cytochrome c oxidase subunit 1) as a standard region. However, as far as plants are concerned, there was no strong consensus on which DNA regions should be used (Fourth International Barcode of Life Conference, www.dnabarcodes2011.org). The Plant Working Group of the Consortium for the Barcode of Life (http://www.barcoding.si.edu/plant_working_group.html) suggested the use of two plastidial coding regions, the *rbcL* and *matK*, as core-barcode for plant identification [14]. Additional regions, such as *trnH-psbA*, could be used for the analysis of closely related taxa [15].

Many DNA barcoding studies on plants analysed the discriminating power of molecular data within relatively homogeneous groups, such as families or genera [14], [15]. In this paper, we discuss the performance of core-barcode region, plus the additional *trnH-psbA* region, in the identification of vascular plants belonging to a local flora of a few hundred species, that of Mt. Valerio (Trieste, NE Italy). This flora is strongly heterogeneous, since it includes one or a few species only for each genus. The DNA barcoding approach is compared to the use of a digital identification key based upon morphological features. The added value of DNA barcoding data to the identification keys produced by FRIDA is discussed, detailing the idea of an innovative integrated identification system, obtained by joining morphological and molecular data.

## Results

### DNA barcoding markers and their performance in plant identification

A group of 50 randomly selected taxa was used to evaluate the intraspecific genetic variability for the three markers. The results of this preliminary survey are reported in Table S2. Amplification and sequencing success were achieved for all samples except *Hieracium racemosum* for *rbcL*, *Koeleria lobata* for *matK*, and *Cistus salvifolius*, *Hieracium racemosum*, and *Stellaria media* for *psbA-trnH*. The *rbcL* sequences showed an averaging complete intraspecific identity for all but six species with a maximum of 0.7% for *Inula hirta*. The *matK* and *trnH-psbA* showed a certain degree of intraspecific variability, but K2P values were consistently lower than 2%, as was expected in the case of a strongly heterogeneous local flora. Based on the reduced intraspecific variability for the three markers, and since this work was not meant to generate alpha-taxonomy, we hereafter conducted our analyses on one sample for each species. This strategy was used to define a local DNA barcoding library to evaluate the discriminating power of the tested markers in the plant identification processes.

High quality and good yield of DNA (from 30 to 50 ng/µl) was obtained from all 347 samples, but 4 species: *Inula spiraeifolia*, *Genista germanica*, *Trifolium arvense* subsp. *arvense*, and *Calamintha nepeta*. For the latter, electrophoretic analysis showed partially degraded DNA in the 100–1000 bp range and low yield of DNA extraction (data not shown). As a consequence, marker-specific DNA barcoding libraries were defined on a total of 343 taxa. The *rbcL* was successfully amplified and sequenced in ca. 98% of the tested samples, and *trnH-psbA* and *matK* in ca. 94% of the samples. However, the latter required three different sets of primers (Table 1). Accession numbers for each DNA barcoding sequence are provided in Table S1. On the whole, a total of 323 sequences for *matK*, 337 sequences for *rbcL* and 323 sequences for *trnH-psbA* were submitted to Genbank as ‘standard barcode’. In our DNA library, one sequence for at least one of the three DNA markers was obtained from all samples, while at least two markers were sequenced from 304 samples (88.6%). The amplification and sequencing of all three markers was obtained for 300 samples (87.5%, see Table 1).

**Table 1:** List of primer pairs and PCR annealing temperatures used in the present study for the three selected DNA barcoding markers.

Locus	Code	Primer name	Sequences (5′-3′)	Annealing temperature	Reference
*rbcL*	-	1F	ATGTCACCACAAACAGAAAC	50°C	[42]
		724R	TCGCATGTACCTGCAGTAGC		[42]
*matK* *	A	390F	CGATCTATTCATTCAATATTTC	53°C	[43]
		1326R	TCTAGCACACGAAAGTCGAAGT		[43]
	B	XF	AATTTACGATCAATTCATTC	50°C	[44]
		5R	GTTCTAGCACAAGAAAGTCG		[44]
	C	1R_KIM	CGTACAGTACTTTTGTGTTTACGAG	55°C	Ki-Joong Kim, unpublished
		3F_KIM	ACCCAGTCCATCTGGAAATCTTGGTTC		Ki-Joong Kim, unpublished
*trnH-psbA*	-	psbA	GTTATGCATGAACGTAATGCTC	53°C	[24]
		trnH	CGCGCATGGTGGATTCACAAATC		[24]

* each pair of primers was used according to [36].

K2P molecular distance (converted into percentage), was used to evaluate the discriminating power of the three DNA barcoding markers in the total flora and in 8 congeneric groups (G1–8). For each group, morphological characters necessary to achieve a correct identification by using the digital key were also reported (Table 2). Between the two core barcode markers the highest genetic variability was observed for *matK*, with a mean K2P value of 27.9% (34.1% for primer copy A, 21% for B and 28.1% for C, data not shown) computed on 323 samples, which lead to the definition of 313 Molecular Operational Taxonomic Units (MOTUs) (data not shown). In the 8 congeneric groups, K2P values ranged from 0.4% (Gr5) to 5.2% (Gr2). Complete sequence identity was observed for some taxa of Gr5 (*Prunus spinosa* subsp. *spinosa*, *P. cerasifera var. cerasifera* and *P. cerasifera* var. *pissardii*), Gr6 (*Senecio vulgaris* and *S. inaequidens*) and Gr7 (*Solanum villosum* subsp. *alatum* and *S. nigrum*).

**Table 2:** Results of DNA barcoding analysis performed for the 8 considered congeneric groups.

Group	N	MOTU (% K2P)					Morphological distinctive traits			
		*rbcL*	*matK*(A)	*matK*(B)	*matK*(C)	*trnH-psbA*	Plant	Leaves	Flowers	Fruits
Gr1- *Acer*	4	3 (0.1)			3 (0.5)	4 (2.0)		Nr. of leaf-lobes, leaf-length	form of inflorescence	angle between the two wings of the fruit
Gr2- *Euphorbia*	6	5 (0.8)	6 (5.2)			6 (20.6)		Form of leaves	shape of glands in the inflorescence	
Gr3-*Geranium*	4	3 (1.3)	4 (3.7)			4 (12.2)		Form of leaves	shape and length of petals	
Gr4-*Medicago*	4	2 (0.7)			3 (2.6)	4 (17.7)			colour of flowers	form of the legume
Gr5-*Prunus*	8	3 (0.2)	6 (0.4)			6 (1.2)	Evergreen vs. deciduous	shape of leaves,	presence-absence of thorns	fruit hairy-hairless
Gr6-*Senecio*	4	3 (0.7)		3 (2.6)		3 (16.4)		Form and hairiness of leaves	presence/absence of ligulate flowers	
Gr7-*Solanum*	4	3 (0.7)			3 (0.5)	3 (3.0)	Habitus of the plant	form of leaves (compound vs. simple),	colour of petals	colour of fruits
Gr8-*Trifolium*	5	5 (0.9)			5 (3.0)	5 (5.9)	plant annual/perennial		Colour of flowers; shape of inflorescence (globose vs. cylindrical)	

Numbers of MOTU and genetic distance values (K2P%) were described for each DNA barcoding regions (in the case of *matK* region data were reported separately for each primer copy).

The discriminated morphological traits used in the FRIDA digital keys to identify the plant species of each group were also described.

N = Number of samples; MOTU: Molecular Operational Taxonomic Unit.

The *rbcL* sequence showed the lowest genetic variability, with 308 MOTUs on 337 amplified sequences. The average genetic distance on total flora was 10.8%. Complete sequence identity was observed in Gr1 (*Acer campestre* and *A. pseudoplatanus*), Gr2 (*Euphorbia maculata* and *E. nicaeensis* subsp. *nicaeensis*), Gr3 (*Geranium sanguineum* and *G. molle*), Gr4 (*Medicago lupulina* and *M. minima*; *M. falcata* subsp. *falcata* and *M. sativa*), Gr6 (*Senecio vulgaris* and *S. inaequidens*), and Gr7 (*Solanum villosum* subsp. *alatum* and *S. nigrum*). K2P distances in Gr5 (*Prunus* samples) were lower than 1%, and five accessions were indistinguishable (Tab. 2).

The amplification of *trnH-psbA* marker produced 318 MOTUs on 322 amplified samples. The high genetic variability did not permit a complete alignment in all taxa. Genetic differences were the greater, ranging from 1.2% (Gr5) to 20.6% (Gr2). Few taxa in Gr5 (*Prunus cerasifera* var. *pissardii* and *P. cerasifera var. cerasifera*; *P. spinosa* subsp. *spinosa* and *P. domestica* subsp. *insititia*), Gr6 (*Senecio vulgaris* and *S. inaequidens*) and Gr7 (*Solanum nigrum* and *S. villosum subsp*. alatum) had identical *trnH-psbA* sequences.

### DNA barcoding support to digital identification keys

On the basis of the three simulated scenarios, the digital key returned three groups of 37, 105 and 41 species respectively, which are undistinguishable due to missing characters in the specimens (see Table S1). Table 3 shows how DNA barcoding data improves the identification success by overcoming the absence of some seasonal morphological features. The use of *rbcL* alone (i.e. the most universal and less variable among the three DNA barcode markers) can reduce the uncertainty in the identification process, leading to the identification of a minimum of 92.7% (Scenario C), up to 95.0% (Scenario B) of the species. *matK* identifies from 95.0% (Scenario C) to 98.0% (Scenario B) of the species, while *trnH-psbA* has the highest values of discrimination (up to 100%). For this marker, MOTUs always correspond to the related taxa in two out of three scenarios.

**Table 3:** Discriminating performance of the three tested DNA barcode markers (*rbcL*, *matK* and *trnH-psbA*) in the three scenarios (S) depicted by FRIDA digital key identification processes on the flora of Mt. Valerio.

S	GP	*rbcL*			*matK*			*trnH-psbA*			*rbcL*+*matK*			*rbcL*+*matK*+*trnH-psbA*		
		N	MOTU	%	N	MOTU	%	N	MOTU	%	N	MOTU	%	N	MOTU	%
A	37	36	34	94.4	35	34	97.1	33	33	100	35	34	97.1	32	32	100
B	105	101	96	95.0	98	96	98.0	100	99	99	96	94	97.9	93	92	98.9
C	41	41	38	92.7	40	38	95.0	37	37	100	40	38	95.0	36	36	100

A full list of selected plants is available in Table S1.

For each group of plants (GP) the number of sequences obtained (N) and the number of Molecular Operational Taxonomic Units (MOTUs) were listed for each marker and their combinations. Based on these values the discriminatory efficiency was calculated as percentage of correctly identified species (%).

The use of the core barcode markers or their combination with *trnH-psbA* did not improve the discriminating success with respect to the use of the last marker alone (Table 3).

As expected, the use of the DNA barcoding does not discriminate among some congeneric species of groups B, *Solanum villosum* subsp. *alatum* and *S. nigrum*.

## Discussion

### DNA barcoding and identification of plants

The variability of *rbcL*, *matK* and *trnH-psbA* sequences can be used to identify most of the plants occurring in the area of Mt. Valerio. Ca. 96% can be distinguished by combining the two core-barcode markers *rbcL* and *matK*. Similar results were reported in previous studies conducted in different areas [15]. [16] reported that the use of *rbcL*+*matK* permits to identify 92% of the woody species in a plot of 50 ha in the tropical forest of Panama. [17] identified 92.7% of the plants of the Koffler Scientific Reserve (Ontario, Canada), using the same markers. Thus, in relatively restricted areas, where a reduced number of closely related species is present [15], [17], as in our case, the combination of *rbcL*+*matK* is effective in identifying plant species.

However, there exist some constraints to the use of the two core-barcode markers. The *matK* gene is considered a good DNA barcode region because it is rapidly evolving [18], but its amplification requires several combinations of primers (3 in this study). As recently discussed at the Fourth International Barcode of Life Conference (www.dnabarcodes2011.org), the *matK* amplification system requires some improvements (i.e. the definition of clade-specific primers, or the identification of universal combinations of primers), in order to be effective when applied as a universal DNA barcode region for plants. On the other hand, the *rbcL* marker, which is easy to amplify, sequence and align, has a limited discrimination power, especially when among closely related species. These results are in agreement with the Fourth International Barcode of Life Conference (www.dnabarcodes2011.org/), during which *matK* and *rbcL* coding regions were, in any case, confirmed as universal core-barcodes.

As stated by other investigators [15], we support the use of the *trnH-psbA* region as an additional marker, especially when DNA barcoding is applied to closely related plant taxa. This region has highly conserved PCR priming sites, and a non-coding region with high numbers of substitutions. Hence, *trnH-psbA* can be a suitable marker to discriminate among closely related species. Although previous research reported the frequent occurrence of stutter PCR products for *trnH-psbA* due to mononucleotide repeats [19], recent technical advancements (i.e. appropriate polymerases; ideal PCR conditions see [20]) have suggested that these problems could be easily overcome.

In addition, as pointed out by [15], a complete exploration of plastidial non-coding markers (particularly *trnH-psbA*) could be useful to decide whether to incorporate them into core-barcode when dealing with plants.

A practical result of our investigation concerns the sampling strategy adopted to develop a DNA barcoding database useful for plant identification. In the local context, the low genetic intraspecific variability suggests that a DNA barcoding profile for only one individual per species is enough “to assist in the process of identifying unknown specimens to known species” [15]. We are aware that this strategy is not suitable for a classical alpha-taxonomy investigation, where a deep sampling coverage is necessary, but it is appropriate in our context where it was essential to characterize the local plant genetic profiles in order to identify unknown specimens, as also suggested by [17].

### Integrated taxonomic identification system

Some authors suggested a superiority of molecular tools in comparison with “classic” identification keys based on morphological data [21]–[22]. In our opinion, however, molecular and morphological data should not be seen as colliding worlds, but as different solutions to a common problem. In some cases DNA barcoding is not successful in discriminating among species, which are closely related phylogenetically, as shown here and in several other studies [23]–[25]. Problems in achieving species identification by using DNA barcoding alone were reported within several angiosperm families, such as Orchidaceae [26], Ericaceae [27] and Lamiaceae [25]. DNA barcoding markers require a certain “evolutionary distance” among the taxa, in order to be used in their identification [14].

Morphology can be useful to discriminate among closely related taxa, as shown in Table 2. Morphological differences could derive from one or few DNA mutations [28]–[29] or from epigenetic variation [30], which could be not detected by DNA barcoding. However, identification keys based upon morphology could be difficult to use when some features are not visible, as happens when specimens are not well developed, or outside specific life stages (e.g. flowering period). For this reason, matrix-based digital keys are usually equipped with multi-entry or multi-access query interfaces [31], which do not force users to follow a fixed sequence of characters in the identification process. Also in these cases, however, the absence of some morphological features could make the identification impossible. In this case, the use of DNA barcoding could complete the identification process by compensating the limits of the morphological approach as clearly showed in our simulations.

While identification keys based on morphology discriminate among morphospecies, DNA barcoding discriminates among MOTUs, and sometimes these two “entities” could not match [32]. In this study, some specimens which showed the same DNA barcode sequences (e.g. *Prunus spinosa* subsp. *spinosa* and *P. domestica* subsp. *insititia*) were readily distinguished by morphological features, while species belonging to the genera *Mentha* (i.e. *M. longifolia* and *M. pulegium* subsp. *pulegium*), and *Inula* (i.e. *I. hirta* and *I. spiraeifolia*) showed similar morphological features, but were successfully distinguished by DNA barcoding. The integration of the two approaches, with the development of a multi-parametric identification system, may enhance the overall effectiveness, and represent a real advancement in plant identification. Such a system could be used in very different scenarios, from the identification of plant parts [33]–[35], to floristic researches and the discovery of new taxa [24].

Software such as FRIDA has the potential to integrate different data sources, including the capacity of using ‘filters’ to automatically reduce a large key by retaining only subsets of species which share a certain character. The inclusion of molecular characters as ‘filters’ does not present any technical problem. In an integrated system, DNA barcoding data could be used as a ‘filter’ by the software which produces and manages the interactive morphological keys. When molecular data are not sufficient to identify a plant at species level, the system will automatically produce a morpho-anatomical key only to the species which are not distinguished by barcode data. This would be an innovative approach to digital identification, which combines morphological and molecular data, overcoming the limits of both approaches, with the potential of becoming the core of a standardised protocol useful in biodiversity surveys, as a new integrated plant identification system, as already proposed by [22], in the so-called Automated Identification Technology (AIT). Moreover, thanks to the available platforms including laptops and mobile phones, these resources could be easily accessible to society-at-large to identify plants, as shown by the KeyToNature initiative (http://www.keytonature.eu/wiki/).

## Materials and Methods

### Experimental design

The area of Mt. Valerio (Trieste, NE Italy), which hosts a well-known flora, was selected to create a digital identification key and a DNA barcoding library. To investigate how molecular-based data could improve the effectiveness of a digital key, a two-step pipeline was followed. In the first step, the universality of three DNA barcode markers (i.e. *matK*, *rbcL* and *trnH-psbA*), the extent of their intraspecific variability, and their discriminating power on a subset of congenerics were investigated. In the second step, the performance of each marker (or of different combinations of them) was quantitatively estimated in three simulated scenarios in which the digital key could fail.

### Survey area

Mt. Valerio is a low hill (215 m) in the north-east suburban area of Trieste. It is characterized by a submediterrean climate, strongly influenced by the Adriatic Sea, with a dry summer, rainy autumn and spring, and occasional frosts in winter. Average annual precipitation is 1016,9 mm, and average annual temperature is 14,1°C. Prevailing winds are the cold and dry Bora, blowing in winter from east-north-east, and the southern, mild and humid Scirocco. The substratum is Flysch, a base-rich formation of sandstones and marls. The whole survey area has a surface of ca. 0,25 km^2^, and includes both highly and semi-natural sites with different types of vegetation. Small oak stands with *Quercus petraea* subsp. *petraea* and *Q. pubescens* subsp. *pubescens* cover the western and southern sides of the hill. A stand of *Carpinus orientalis* subsp. *orientalis* lies on the more humid western side, while an artificial pine wood of *Pinus nigra* subsp. *nigra* covers the northern and eastern slopes. Shrublands with *Spartium junceum* and more or less close formations of *Robinia pseudoacacia* are located mostly on the south-eastern slopes.

### The digital key

The digital key to the flora of Mt. Valerio was generated by FRIDA, on the basis of morpho-anatomical, ecological and distributional data, plus original images and drawings, deriving from a floristic list by Poldini et al. (unpublished data). The key is freely accessible online at the address http://dbiodbs.units.it/carso/chiavi_pub21?sc=77, in Italian and English. It can be used with two query interfaces [7]: 1) single-access (dichotomous), which requires the choice between two states of a character at each step of the identification process, and 2) multi-entry, which allows the use of several characters at the same time, hence strongly reducing the list of organisms in the first step of the identification process, after which the identification continues with a dichotomous interface for the remaining species. At the end of the identification, a taxon page appears, with scientific name, description and images, which are useful as “visual census”, to verify the correctness of the identification.

### Plant specimens for DNA analysis

A total of 347 species were sampled in the period 2009–2010. For each individual, young leaves or buds were collected from at least three different individuals, and stored at −20°C. All samples were vouchered as ‘MIB:ZPL’ following the protocol specified by the biorepositories initiative (www.biorepositories.org), and the data standards for BARCODE Records in INSDC (http://barcoding.si.edu/PDF/DWG_data_standards-Final.pdf). All experiments, procedures and ethical issues were conformed to the competent national ethical bodies. No specific permits were required for sampling activities, which were conducted in a non protected area, in accordance with the national and regional laws. The location was not privately owned or in any way protected and field studies did not involve endangered or protected species. Specimens and voucher codes are listed in Table S1.

A total of 100 mg of plant material was used for DNA extraction. Genomic DNA was isolated using the DNeasy Isolation and Purification kit (Qiagen, Hilden, Germany), to obtain high-quality DNA, free of polysaccharides or other metabolites that might interfere with DNA amplification [34].

### DNA Barcoding analysis

DNA barcoding analysis was performed with three different DNA markers; the *rbcL* and *matK* coding regions and the non-coding *trnH-psbA* intergenic spacer of plastidial DNA. PCR amplification was performed by using puReTaq Ready-To-Go PCR beads (Amersham Bioscience, Freiburg, Germany) in a 25 µL reaction according to the manufacturer's instructions. PCR cycles consisted of an initial denaturation step for 7 min at 94°C, 35 cycles of denaturation (45 s at 94°C), annealing (30 s at different temperatures; see Table 1), and extension (1 min at 72°C), and a final extension at 72°C for 7 min. One universal primer pair was used for the amplification of *rbcL* and *trnH-psbA*, while three different combinations of primers were used for the amplification of *matK*, as suggested by [36]. Further details on primers and conditions of amplification are provided in Table 1. PCR products were bidirectionally sequenced by using an ABI 155 3730XL automated sequencer at Macrogen Inc., Korea. Manual editing of raw traces and subsequent alignments of forward and reverse sequences allowed to assign sequences to almost all the species. The 3′ and 5′ terminals were clipped to generate consensus sequences for each *taxon*. In order to avoid the inclusion of inadvertently amplified nuclear pseudogenes of plastidial origin (see [37]), barcode sequences were checked following the guidelines proposed by [38]–[39]. The sequences have been deposited in the EMBL Data Library.

To verify the universality of the three DNA barcode regions, the markers were routinely amplified and sequenced in the highest number. Only the most universal primer combinations for each candidate marker were tested (Table 1). For all taxa and loci, PCR amplifications in a two-stage trial were made. In the first stage, standard PCR conditions described above were used, starting from 10 ng of DNA template. Samples which were not amplified or which produced multiple or nonspecific PCR products were amplified again under less stringent conditions, by reducing the annealing temperature of 5°C, and executing 40 PCR cycles. In case of further failures, PCR products of both stages were amplified again by using 1 and 20 ng of DNA template. Any further negative result was considered a failure, and the corresponding samples were removed from the dataset.

To evaluate the intraspecific genetic variability of the markers, a total of three individuals for 50 randomly selected species were analysed (see Table S2). The performance of each marker was also evaluated by taking into account its resolution power on the total flora, and on 8 congeneric groups of strictly related taxa:

Gr1: Specimens of four species of *Acer*: *A. campestre*, *A. monspessulanum* subsp. *monspessulanum*, *A. negundo* and *A. pseudoplatanus*.
Gr2: Specimens of five species of *Euphorbia*: *E. characias* subsp. *wulfenii*, *E. cyparissias*, *E. helioscopia*, *E. maculata*, *E. nicaeensis* subsp. *nicaeensis* and *E. peplus*.
Gr3: Specimens of four species of *Geranium*: *G. columbinum*, *G. molle*, *G. purpureum* and *G. sanguineum*.
Gr4: Specimens of four species of *Medicago*: *M. falcata* subsp. *falcata*, *M. lupulina*, *M. minima* and *M. sativa*.
Gr5: Specimens of eight species and subspecies of *Prunus*: *P. avium* subsp. *avium*, *P. cerasifera*, *P. cerasifera var. pissardii*, *P. domestica* subsp. *insititia*, *P. laurocerasus*, *P. mahaleb*, *P. persica* and *P. spinosa* subsp. *spinosa*.
Gr6: Specimens of four species of *Senecio*: *S. gibbosus*, *S. inaequidens*, *S. jacobaea* and *S. vulgaris*.
Gr7: Specimens of four species of *Solanum*: *S. dulcamara*, *S. lycopersicum*, *S. nigrum* and *S. villosum* subsp. *alatum*.
Gr8: Specimens of six species of *Trifolium*: *T. arvense*, *T. campestre*, *T. montanum* subsp. *montanum*, *T. pretense* subsp. *pratense*, *T. repens* and *T. rubens*.

For each group and for each tested marker, DNA barcode sequences were aligned using MUSCLE – default options [40]. According to the guidelines provided by the Consortium for the Barcoding of Life (http://www.barcoding.si.edu/protocols.html), levels of genetic variation were analyzed by using MEGA 4.0 [41], to generate Kimura 2-parameter (K2P) distance matrices for each locus. Taxa which showed complete identity of DNA barcode sequence were considered non-identifiable with the marker under examination.

### Three hypothetical scenarios from the digital key

To simulate situations in which a digital identification key based on morphological features could fail in achieving correct species identification, three different scenarios were defined. A description is given for each scenario, together with the list of characters used in the digital identification key (multi-entry query interface). In all cases, the identification, which should continue with the dichotomous query interface, fails because of missing features on the specimen. The simulations use a special version of the digital key containing only those taxa for which at least a sequence was successfully amplified.

Scenario A – The simulation takes into account a specimen of a non laticiferous, terrestrial herbaceous plant with opposite, non-whorled, entire leaves, collected out of its flowering period. Characters used in the multi-entry query interface of the digital key are: 1) herbaceous plant or a small shrub (<50 cm), 2) green, with chlorophyll, 3) without spines, 4) terrestrial, 5) leaves opposite, 6) leaves simple, 7) leaves not whorled.

The result from the multi-entry query interface is a list of 37 taxa, the identification of which can continue by using the dichotomous query interface, which asks whether the plant is laticiferous or not (answer: no) and then whether flowers have petals or not. The identification process, at this point, cannot be continued, because the specimen does not have flowers.

Scenario B - The simulation takes into account a specimen of a non laticiferous, terrestrial herbaceous plant with alternate, simple leaves, collected out of its flowering period. Characters used in the multi-entry query interface of the digital key are: 1) herbaceous plant or a small shrub (<50 cm), 2) green, with chlorophyll, 3) without spines, 4) terrestrial, 5) with leaves, 6) leaves not opposite, 7) leaves entire, 8) leaves not whorled

The result from the multi-entry query interface is a list of 105 taxa. The dichotomous query interface asks whether the flowers have petals or not. The identification process, at this point, cannot be continued, because the specimen does not have flowers.

Scenario C - The simulation takes into account a specimen of a tree collected in late Autumn, with dry fruits but missing leaves. Characters used in the multi-entry query interface are: 1) tree, woody climber or shrub >50 cm tall, 2) not a woody climber, 3) deciduous, 4) terrestrial, 5) fruit dry.

The result from the multi-entry query interface is a list of 41 taxa. The identification continues with the dichotomous interface, which asks whether the leaves are opposite or not. The identification process, at this point, cannot be continued, because the specimen does not have leaves.

## Supporting Information

Table S1 List of the analysed plants collected from Mt. Valerio flora. For each sample the voucher number (V.N.), the species name (Nomenclature follows [45], [46]) and the Accession Numbers corresponding to DNA sequences of the three analysed markers are also included; “-”: sequencing failure. To evaluate the contribution of intraspecific variability, three specimens (i–iii) belonging to 50 randomly selected species, were analysed with the three DNA barcoding markers. Plant species included in the three independent FRIDA digital key simulations (scenarios A,B,C) were also shown (x).
(DOC)

Table S2 Evaluation of intraspecific genetic variability. For a subset of 50 plant species from the Mt. Valerio the mean values of intraspecific variability and the standard error for the three tested makers are provided. Sampling details can be retrieved from Table S1.
(DOCX)
