# Supplementary material for: DNA Barcoding as an Effective Tool in Improving a Digital Plant Identification System: A Case Study for the Area of Mt. Valerio, Trieste (NE Italy)
Source: PLoS One. 2012 Sep 10;7(9):e43256. doi: 10.1371/journal.pone.0043256 (PMC3438168; doi:10.1371/journal.pone.0043256)
Supplement: Table S1 — List of the analysed plants collected from Mt. Valerio flora. For each sample the voucher number (V.N.), the species name (Nomenclature follows [45], [46]) and the Accession Numbers corresponding to DNA sequences of the three analysed markers are also included; “-”: sequencing failure. To evaluate the contribution of intraspecific variability, three specimens (i–iii) belonging to 50 randomly selected species, were analysed with the three DNA barcoding markers. Plant species included in the three independent FRIDA digital key simulations (scenarios A,B,C) were also shown (x). (DOC) [file pone.0043256.s001.doc]

**Table S1.** List of the analysed plants collected from Mt. Valerio flora. For each sample the voucher number (V.N.), the species name (Nomenclature follows [45, 46]) and the Accession Numbers corresponding to DNA sequences of the three analysed markers are also included; "-": sequencing failure.

To evaluate the contribution of intraspecific variability, three specimens (i-iii) belonging to 50 randomly selected species, were analysed with the three DNA barcoding markers.

Plant species included in the three independent FRIDA digital key simulations (scenarios A,B,C) were also shown (x).

| **V.N.** | **Species Name** | **Accession Number** | | | **Scenario** | | |
| --- | --- | --- | --- | --- | --- | --- | --- |
|  |  | ***rbcL*** | ***matK*** | ***trnH-psbA*** | **A** | **B** | **C** |
| MIB:ZPL:03544 | *Acanthus mollis* L. subsp. *mollis* | HE963302 | HE967332 | HE966457 |  |  |  |
| MIB:ZPL:03042 | *Acer campestre* L*.* | HE963303 | HE970664 | HE966458 |  |  | x |
| MIB:ZPL:03541 | *Acer monspessulanum* L. subsp*. monspessulanum* | HE963304 | HE967333 | HE966459 |  |  | x |
| MIB:ZPL:03397 | *Acer negundo* L. | HE963305 | HE967334 | HE966460 |  |  | x |
| MIB:ZPL:03188 | *Acer pseudoplatanus* L. | HE963306 | HE967335 | HE966461 |  |  | x |
| MIB:ZPL:03199 | *Achillea collina* (Becker ex Wirtg.) Heimerl | HE963307 | HE967336 | HE966462 |  |  |  |
| MIB:ZPL:03192 | *Aesculus hippocastanum* L. | HE963308 | HE967337 | HE966463 |  |  | x |
| MIB:ZPL:03548 | *Ailanthus altissima* (Mill.) Swingle | HE963309 | HE967338 | - |  |  | x |
| MIB:ZPL:03658 | *Aira elegantissima* Schur subsp*. elegantissima* | HE963310 | HE966874 | HE966464 |  | x |  |
| MIB:ZPL:03258 | *Alliaria petiolata* (M. Bieb.) Cavara & Grande | HE963311 | HE967339 | HE966465 |  | x |  |
| MIB:ZPL:03206 | *Allium carinatum* L. | HE963312 | - | HE966466 |  | x |  |
| MIB:ZPL:03552 | *Allium vineale* L. | HE963313 | HE966875 | HE966467 |  | x |  |
| MIB:ZPL:03998 | *Amaranthus retroflexus* L. | HE963314 | HE967341 | HE966469 |  | x |  |
| MIB:ZPL:03244 | *Amaranthus deflexus* L. | HE963315 | HE967340 | HE966468 |  | x |  |
| MIB:ZPL:04000(i-iii) | *Ambrosia artemisiifolia* L. | HE963316  HE963317  HE963318 | HE970665  HE970666  HE970667 | HE966470  HE966471  HE966472 |  |  |  |
| MIB:ZPL:03191 | *Anagallis arvensis* L. subsp. *arvensis* | HE963319 | HE966876 | HE966473 | x |  |  |
| MIB:ZPL:03666 | *Anthericum ramosum* L. | - | - | HE966474 |  | x |  |
| MIB:ZPL:03068 | *Anthoxanthum odoratum* L. subsp. *odoratum* | HE963320 | HE966877 | HE966475 |  | x |  |
| MIB:ZPL:03988 | *Antirrhinum majus* L. subsp. *majus* | HE963321 | HE967342 | HE966476 | x |  |  |
| MIB:ZPL:03083 | *Arabis sagittata* (Bertol.) DC. | HE963322 | - | HE966477 |  | x |  |
| MIB:ZPL:03553(i-iii) | *Arctium minus* (Hill) Bernh. | HE963323  HE963324  HE963325 | HE970668  HE970669  HE970670 | HE966478  HE966479  HE966480 |  | x |  |
| MIB:ZPL:03209(i-iii) | *Arenaria serpyllifolia* L. subsp. *serpyllifolia* | HE963326  HE963327  HE963328 | HE967343  HE967344  HE967345 | HE966481  HE966482  HE966483 | x |  |  |
| MIB:ZPL:03254(i-iii) | *Aristolochia clematitis* L. | HE963329  HE963330  HE963331 | HE967346  HE967347  HE967348 | HE966484  HE966485  HE966486 |  | x |  |
| MIB:ZPL:03071(i-iii) | *Aristolochia lutea* Desf. | HE963332  HE963333  HE963334 | HE970671  HE970672  HE970673 | HE966487  HE966488  HE966489 |  | x |  |
| MIB:ZPL:04516 | *Arrhenatherum elatius* (L.) P. Beauv. ex J. Presl & C. Presl subsp. *elatius* | HE963335 | HE970674 | HE966490 |  | x |  |
| MIB:ZPL:03238 | *Artemisia absinthium* L. | HE963336 | HE970675 | HE966491 |  |  |  |
| MIB:ZPL:03252 | *Artemisia vulgaris* L. | HE963337 | HE967349 | HE966492 |  |  |  |
| MIB:ZPL:04514 | *Arundo donax* L. | HE963338 | HE970676 | HE966493 |  | x |  |
| MIB:ZPL:03038 | *Asparagus acutifolius* L. | - | HE967350 | HE966494 |  | x |  |
| MIB:ZPL:03679 | *Asperula cynanchica* L. subsp. *cynanchica* | - | HE970677 | HE966495 |  |  |  |
| MIB:ZPL:03672 | *Asperula purpurea* (L.) Ehrend. subsp. *purpurea* | HE963339 | HE970678 | HE966496 |  |  |  |
| MIB:ZPL:03051 | *Asplenium adiantum-nigrum* L. subsp. *adiantum-nigrum* | HE963340 | - | HE966497 |  |  |  |
| MIB:ZPL:03557 | *Asplenium ruta-muraria* L. subsp. *ruta-muraria* | HE963341 | - | HE966498 |  |  |  |
| MIB:ZPL:03096 | *Asplenium trichomanes* L. subsp. *quadrivalens* D.E. Mey. | HE963342 | - | HE966499 |  |  |  |
| MIB:ZPL:03995(i-iii) | *Atriplex patula* L. | HE963343  HE963344  HE963345 | HE967351  HE967352  HE967353 | HE966500  HE966501  HE966502 |  | x |  |
| MIB:ZPL:04015 | *Aucuba japonica* Thunb. | HE963346 | HE966878 | HE966503 |  |  |  |
| MIB:ZPL:03241 | *Avena sterilis* L. s.l. | HE963347 | HE966879 | HE966504 |  | x |  |
| MIB:ZPL:03255 | *Ballota nigra* L. subsp*. meridionalis* (Bég.) Bég*.* | HE963348 | HE967354 | HE966505 | x |  |  |
| MIB:ZPL:03201 | *Bellis perennis* L*.* | HE963349 | HE970679 | HE966506 |  | x |  |
| MIB:ZPL:03994(i-iii) | *Berberis thunbergii* DC. | HE963350  HE963351  HE963352 | HE967355  HE967356  HE967357 | HE966507  HE966508  HE966509 |  |  |  |
| MIB:ZPL:04511 | *Bidens subalternans* DC. | HE963353 | HE970680 | HE966510 |  |  |  |
| MIB:ZPL:03527 | *Borago officinalis* L*.* | HE963354 | HE966880 | HE966511 |  |  |  |
| MIB:ZPL:03691 | *Bothriochloa ischaemum* (L.) Keng | HE963355 | HE966881 | HE966512 |  | x |  |
| MIB:ZPL:03076 | *Brachypodium rupestre* (Host) Roem. & Schult. | HE963356 | HE967358 | HE966513 |  | x |  |
| MIB:ZPL:03212 | *Bromus commutatus* Schrad. subsp*. commutatus* | HE963357 | HE966882 | HE966514 |  | x |  |
| MIB:ZPL:03093 | Bromus erectus Huds. subsp*.* erectus | HE963358 | HE966883 | HE966515 |  | x |  |
| MIB:ZPL:03075 | *Bromus sterilis* L*.* | HE963359 | HE966884 | HE966516 |  | x |  |
| MIB:ZPL:04012 | *Broussonetia papyrifera* (L.) Vent. | HE963360 | HE967359 | HE966517 |  |  |  |
| MIB:ZPL:03997 | *Buddleja davidii* Franch. | HE963361 | HE967360 | HE966518 |  |  | x |
| MIB:ZPL:03989 | *Buglossoides purpurocaerulea* (L.) I.M. Johnst. | HE963362 | HE967361 | HE966519 |  | x |  |
| MIB:ZPL:03676 | *Bupleurum praealtum* L. | HE963363 | HE970681 | HE966520 |  |  |  |
| MIB:ZPL:03661 | *Bupleurum veronense* Turra | HE963364 | HE970682 | HE966521 |  |  |  |
| MIB:ZPL:03089 | *Buxus balearica* Lam. | HE963365 | HE966885 | HE966522 |  |  |  |
| MIB:ZPL:04006(i-iii) | *Buxus sempervirens* L. | HE963366  HE963367  HE963368 | HE966886  HE966887  HE966888 | HE966523  HE966524  HE966525 |  |  |  |
| MIB:ZPL:03564 | Calamintha nepeta (L.) Savi | - | - | - | x |  |  |
| MIB:ZPL:03981 | *Callicarpa americana* L. | HE963369 | HE967362 | HE966526 |  |  |  |
| MIB:ZPL:03062(i-iii) | *Calluna vulgaris* (L.) Hull | HE963370  HE963371  HE963372 | HE966889  HE966890  HE966891 | HE966527  HE966528  HE966529 |  |  |  |
| MIB:ZPL:03566 | *Calycanthus floridus* L. | HE963373 | HE970683 | HE966530 |  |  | x |
| MIB:ZPL:03194 | *Campanula rapunculus* L. | HE963374 | HE970684 | HE966531 |  | x |  |
| MIB:ZPL:03224 | *Campanula trachelium* L. subsp*. trachelium* | HE963375 | HE970685 | HE966532 |  | x |  |
| MIB:ZPL:03185 | *Capsella bursa-pastoris* (L.) Medik*. subsp. bursa-pastoris* | HE963376 | HE967363 | HE966533 |  | x |  |
| MIB:ZPL:04002(i-iii) | *Cardamine hirsuta* L. | HE963377  HE963378  HE963379 | HE967364  HE967365  HE967366 | HE966534  HE966535  HE966536 |  |  |  |
| MIB:ZPL:03195(i-iii) | *Carex flacca* Schreb. subsp*. flacca* | HE963380  HE963381  HE963382 | HE966892  HE966893  HE966894 | HE966537  HE966538  HE966539 |  | x |  |
| MIB:ZPL:03112(i-iii) | *Carex pairae* F.W. Schultz | HE963383  HE963384  HE963385 | HE966895  HE966896  HE966897 | HE966540  HE966541  HE966542 |  | x |  |
| MIB:ZPL:03043 | *Carpinus orientalis* Mill. subsp*. orientalis* | HE963386 | HE966898 | HE966543 |  |  | x |
| MIB:ZPL:03568 | *Castanea sativa* Mill*.* | HE963387 | HE967367 | HE966544 |  |  | x |
| MIB:ZPL:03983(i-iii) | *Catalpa bignonioides* Walter | HE963388  HE963389  HE963390 | HE967368  HE967369  HE967370 | HE966545  HE966546  HE966547 |  |  | x |
| MIB:ZPL:03567(i-iii) | *Catalpa speciosa* (Warder) Engelm*.* | HE963391  HE963392  HE963393 | HE967371  HE967372  HE967373 | HE966548  HE966549  HE966550 |  |  | x |
| MIB:ZPL:03108 | *Cedrus deodara* (Roxb.) G. Don | HE963394 | HE966899 | HE966551 |  |  |  |
| MIB:ZPL:03095 | *Celtis australis* L. subsp*. australis* | HE963395 | HE967374 | HE966552 |  |  |  |
| MIB:ZPL:03246 | *Centaurea cristata* Bartl. | HE963396 | HE970686 | HE966553 |  |  |  |
| MIB:ZPL:03377 | *Centaurea jacea* L. subsp*. weldeniana* (Rchb.) Greuter | HE963397 | HE970687 | HE966554 |  | x |  |
| MIB:ZPL:03533 | *Cercis siliquastrum* L. subsp*. siliquastrum* | HE963398 | - | HE966555 |  |  | x |
| MIB:ZPL:03680 | *Cervaria rivinii* Gaertn*.* | HE963399 | HE970688 | HE966556 |  |  |  |
| MIB:ZPL:03225 | *Ceterach officinarum* L. s.l*.* | HE963400 | - | HE966557 |  |  |  |
| MIB:ZPL:03239 | *Chaenorhinum minus* (L.) Lange subsp*. minus* | HE963401 | HE966900 | HE966558 | x | x |  |
| MIB:ZPL:03183(i-iii) | *Chaerophyllum temulum* L. | HE963402  HE963403  HE963404 | HE967375  HE967376  HE967377 | HE966559  HE966560  HE966561 |  |  |  |
| MIB:ZPL:04004 | *Chamaecyparis lawsoniana* (A.Murray) Parl. | HE963405 | HE966901 | HE966562 |  |  |  |
| MIB:ZPL:03574 | *Chamaesyce maculata* (L.) Small (syn. *Euphorbia maculata* L.) | HE963406 | HE966902 | HE966563 |  |  |  |
| MIB:ZPL:03387 | *Chelidonium majus* L. | HE963407 | HE967378 | HE966564 |  |  |  |
| MIB:ZPL:03264 | *Chenopodium album* L. subsp*. album* | HE963408 | HE967379 | HE966565 |  | x |  |
| MIB:ZPL:03996(i-iii) | *Chenopodium ambrosioides* L. | HE963409  HE963410  HE963411 | HE967380  HE967381  HE967382 | HE966566  HE966567  HE966568 |  | x |  |
| MIB:ZPL:03690 | *Chondrilla juncea* L. | HE963412 | HE970689 | HE966569 |  | x |  |
| MIB:ZPL:03229 | *Chrysopogon gryllus* (L.) Trin. | HE963413 | HE967383 | HE966570 |  | x |  |
| MIB:ZPL:03382 | *Cichorium intybus* L*.* | HE963414 | HE970690 | HE966571 |  |  |  |
| MIB:ZPL:03665(i-iii) | *Cirsium vulgare* (Savi) Ten. subsp. *vulgare* | HE963415  HE963416  HE963417 | HE970691  HE970692  HE970693 | HE966572  HE966573  HE966574 |  |  |  |
| MIB:ZPL:03674 | *Cistus monspeliensis* L. | HE963418 | - | - |  |  |  |
| MIB:ZPL:03061(i-iii) | *Cistus salviifolius* L*.* | HE963419  HE963420  HE963421 | HE967384  HE967385  HE967386 | - | x |  |  |
| MIB:ZPL:03102 | *Clematis vitalba* L. | HE963422 | HE967387 | HE966575 |  |  |  |
| MIB:ZPL:03663 | *Clinopodium vulgare* L. subsp*. vulgare* | HE963423 | HE967388 | HE966576 | x |  |  |
| MIB:ZPL:03251 | *Commelina communis* L*.* | HE963424 | HE966903 | HE966577 |  | x |  |
| MIB:ZPL:03396 | Consolida regalis Gray subsp. *regalis* | HE963425 | HE967389 | HE966578 |  |  |  |
| MIB:ZPL:03389 | *Convolvulus arvensis* L. | HE963426 | HE967390 | HE966579 |  | x |  |
| MIB:ZPL:03130(i-iii) | *Cornus sanguinea* L. subsp*. hungarica* (Kárpáti) Soó | HE963427  HE963428  HE963429 | HE966904  HE966905  HE966906 | HE966580  HE966581  HE966582 |  |  |  |
| MIB:ZPL:03535 | *Corylus maxima* Mill*.* | HE963430 | HE967391 | HE966583 |  |  | x |
| MIB:ZPL:03047 | *Cotinus coggygria* Scop*.* | HE963431 | HE966907 | HE966584 |  |  | x |
| MIB:ZPL:03041 | *Crataegus monogyna* Jacq*.* | HE963432 | HE966908 | HE966585 |  |  |  |
| MIB:ZPL:03381(i-iii) | *Crepis neglecta* L. | HE963433  HE963434  HE963435 | HE970694  HE970695  HE970696 | HE966586  HE966587  HE966588 |  | x |  |
| MIB:ZPL:03394(i-iii) | *Crepis setosa* Haller f. | HE963436  HE963437  HE963438 | HE970697  HE970698  HE970699 | HE966589  HE966590  HE966591 |  | x |  |
| MIB:ZPL:04507 | *Cupressus arizonica* Greene | HE963439 | HE966909 | HE966592 |  |  |  |
| MIB:ZPL:03542 | *Cupressus sempervirens* L. | HE963440 | HE966910 | HE966593 |  |  |  |
| MIB:ZPL:03570 | *Cynodon dactylon* (L.) Pers*.* | HE963441 | HE966911 | HE966594 |  | x |  |
| MIB:ZPL:03685 | *Cytisus hirsutus* L. s.l. | HE963442 | HE967392 | - |  |  |  |
| MIB:ZPL:03055 | *Cytisus nigricans* L. subsp*. atratus* (Schur) Nyman | HE963443 | HE967393 | HE966595 |  |  | x |
| MIB:ZPL:03069 | *Dactylis glomerata* L. subsp*. glomerata* | HE963444 | HE967394 | HE966596 |  | x |  |
| MIB:ZPL:03684 | *Datura stramonium* L. subsp*. stramonium* | HE963445 | HE967395 | HE966597 |  | x |  |
| MIB:ZPL:04001 | *Daucus carota* L. subsp*. carota* | HE963446 | HE967396 | HE966598 |  |  |  |
| MIB:ZPL:03659 | *Dianthus monspessulanus* L*.* | HE963447 | HE967397 | HE966599 | x |  |  |
| MIB:ZPL:03122(i-iii) | *Dianthus sylvestris* Wulfen subsp*. tergestinus* (Rchb.) Hayek | HE963448  HE963449  HE963450 | HE967398  HE967399  HE967400 | HE966600  HE966601  HE966602 | x |  |  |
| MIB:ZPL:03671 | *Diospyros kaki* L. f. | HE963451 | HE967401 | HE966603 |  |  |  |
| MIB:ZPL:03689 | *Diospyros lotus* L. | HE963452 | HE967402 | HE966604 |  |  |  |
| MIB:ZPL:03380(i-iii) | *Diplotaxis tenuifolia* (L.) DC*.* | HE963453  HE963454  HE963455 | HE967403  HE967404  HE967405 | HE966605  HE966606  HE966607 |  |  |  |
| MIB:ZPL:03220 | Dorycnium herbaceum Vill. | HE963456 | - | HE966608 |  |  |  |
| MIB:ZPL:03538 | *Echium vulgare* L. subsp*. vulgare* | HE963457 | HE967406 | HE966609 |  | x |  |
| MIB:ZPL:03215 | Elymus hispidus (Opiz) Melderis | HE963458 | HE966912 | HE966610 |  | x |  |
| MIB:ZPL:03261 | *Emerus major* Mill. subsp*. emeroides* (Boiss. & Spruner) Soldano & F. Conti | HE963459 | HE966913 | HE966611 |  |  | x |
| MIB:ZPL:03980 | *Eragrostis pilosa* (L.) P. Beauv. subsp. *pilosa* | HE963460 | HE966914 | HE966612 |  | x |  |
| MIB:ZPL:04506 | *Erica carnea* L. subsp*. carnea* | HE963461 | HE967407 | HE966613 |  |  |  |
| MIB:ZPL:03187(i-iii) | *Erigeron annuus* (L.) Desf. subsp*. annuus* | HE963462  HE963463  HE963464 | HE967408  HE967409  HE967410 | HE966614  HE966615  HE966616 |  | x |  |
| MIB:ZPL:03236(i-iii) | *Erigeron sumatrensis* Retz. | HE963465  HE963466  HE963467 | HE970700  HE970701  HE970702 | HE966617  HE966618  HE966619 |  | x |  |
| MIB:ZPL:03395 | *Eryngium amethystinum* L. | HE963468 | HE967411 | HE966620 |  |  |  |
| MIB:ZPL:03248 | *Euonymus europaeus* L. | HE963469 | HE966915 | HE966621 |  |  | x |
| MIB:ZPL:03686 | *Euonymus japonicus* L. f. | HE963470 | HE967412 | - |  |  |  |
| MIB:ZPL:03529(i-iii) | *Euphorbia characias* L. subsp*. wulfenii* (Hoppe ex Koch) A.R.Sm*.* | HE963471  HE963472  HE963473 | HE966916  HE966917  HE966918 | HE966622  HE966623  HE966624 |  |  |  |
| MIB:ZPL:03056(i-iii) | *Euphorbia cyparissias* L. | HE963474  HE963475  HE963476 | HE966919  HE966920  HE966921 | HE966625  HE966626  HE966627 |  | x |  |
| MIB:ZPL:03203 | *Euphorbia helioscopia* L. | HE963477 | HE966922 | HE966628 |  | x |  |
| MIB:ZPL:03667 | *Euphorbia nicaeensis* All. subsp*. nicaeensis* | HE963478 | HE966923 | HE966629 |  | x |  |
| MIB:ZPL:03210(i-iii) | *Euphorbia peplus* L. | HE963479  HE963480  HE963481 | HE966924  HE966925  HE966926 | HE966630  HE966631  HE966632 |  | x |  |
| MIB:ZPL:04013 | *Fallopia baldschuanica* (Regel) Holub | HE963482 | HE967413 | HE966633 |  | x | x |
| MIB:ZPL:04515 | *Fallopia convolvulus* (L.) Á. Löve | HE963483 | HE967414 | HE966634 |  | x |  |
| MIB:ZPL:03066 | *Ferulago campestris* (Besser) Grecescu | HE963484 | HE967415 | - |  |  |  |
| MIB:ZPL:03058 | *Festuca heterophylla* Lam. | HE963485 | HE966927 | HE966635 |  | x |  |
| MIB:ZPL:03213 | *Festuca rubra* L. subsp*. rubra* | HE963486 | HE966928 | HE966636 |  | x |  |
| MIB:ZPL:03207 | *Ficus carica* L. | HE963487 | HE966929 | HE966637 |  |  |  |
| MIB:ZPL:03660 | *Filago germanica* (L.) Huds*.* | HE963488 | HE970703 | HE966638 |  | x |  |
| MIB:ZPL:03200 | *Filipendula vulgaris* Moench | HE963489 | HE966930 | HE966639 |  |  |  |
| MIB:ZPL:03534 | *Fraxinus angustifolia* Vahl subsp*. oxycarpa* (Willd.) Franco & Rocha Afonso | HE963490 | HE966931 | HE966640 |  |  | x |
| MIB:ZPL:03045 | *Fraxinus ornus* L. subsp*. ornus* | HE963491 | HE966932 | HE966641 |  |  | x |
| MIB:ZPL:03991 | *Galatella linosyris* (L.) Rchb.f. subsp*. linosyris* | HE963492 | HE970704 | HE966642 |  | x |  |
| MIB:ZPL:03383(i-iii) | *Galinsoga quadriradiata* Ruiz & Pav*.* | HE963493  HE963494  HE963495 | HE970705  HE970706  HE970707 | HE966643  HE966644  HE966645 | x |  |  |
| MIB:ZPL:03129 | *Galium aparine* L. | HE963496 | HE966933 | HE966646 |  |  |  |
| MIB:ZPL:03111 | *Galium lucidum* All. subsp*. lucidum* | HE963497 | - | HE966647 |  |  |  |
| MIB:ZPL:03064 | *Genista tinctoria* L*.* | HE963498 | - | HE966648 |  | x |  |
| MIB:ZPL:03059 | *Genista germanica* L. | - | - | - |  |  |  |
| MIB:ZPL:03081 | *Geranium columbinum* L. | HE963499 | HE966934 | HE966649 |  |  |  |
| MIB:ZPL:04512 | *Geranium molle* L. | HE963500 | HE970708 | HE966650 |  |  |  |
| MIB:ZPL:03080 | *Geranium purpureum* Vill*.* | HE963501 | HE966935 | HE966651 |  |  |  |
| MIB:ZPL:03065 | *Geranium sanguineum* L*.* | HE963502 | HE966936 | HE966652 |  |  |  |
| MIB:ZPL:03128 | *Geum urbanum* L. | HE963503 | HE967416 | HE966653 |  |  |  |
| MIB:ZPL:04003 | *Ginkgo biloba* L*.* | HE963504 | HE970709 | HE966654 |  |  |  |
| MIB:ZPL:04513 | *Hedera algeriensis* Hibberd | HE963505 | HE970710 | HE966655 |  |  |  |
| MIB:ZPL:03037(i-iii) | *Hedera helix* L. s.l. | HE963506  HE963507  HE963508 | HE966937  HE966938  HE966939 | HE966656  HE966657  HE966658 |  |  |  |
| MIB:ZPL:03113 | *Helianthemum nummularium* (L.) Mill. subsp. *obscurum* (Čelak.) Holub | HE963509 | HE967417 | HE966659 | x |  |  |
| MIB:ZPL:04011 | *Hibiscus syriacus* L. | HE963510 | HE967418 | HE966660 |  |  | x |
| MIB:ZPL:03084 | *Hieracium pilosella* L. | HE963511 | HE970711 | HE966661 |  | x |  |
| MIB:ZPL:03245 | *Hieracium piloselloides* Vill. | HE963512 | HE970712 | HE966662 |  | x |  |
| MIB:ZPL:04508(i-iii) | *Hieracium racemosum* Waldst. & Kit. ex Willd. | - | HE970713  HE970714  HE970715 | - |  | x |  |
| MIB:ZPL:03078 | *Hordeum murinum* L. subsp*. murinum* | HE963513 | HE966940 | HE966663 |  | x |  |
| MIB:ZPL:04509 | *Hylotelephium maximum* (L.) Holub subsp*. maximum* | HE963514 | HE967419 | HE966664 |  |  |  |
| MIB:ZPL:03678 | *Hypericum perforatum* L. subsp*. perforatum* | HE963515 | HE966941 | HE966665 | x |  |  |
| MIB:ZPL:04014 | *Ilex aquifolium* L. | HE963516 | HE967420 | HE966666 |  |  |  |
| MIB:ZPL:03067(i-iii) | *Inula hirta* L. | HE963517  HE963518  HE963519 | HE970716  HE970717  HE970718 | HE966667  HE966668  HE966669 |  | x |  |
| MIB:ZPL:03555 | *Inula spiraeifolia* L*.* | - | - | - |  | x |  |
| MIB:ZPL:03222 | *Iris foetidissima* L. | HE963520 | HE967421 | HE966670 |  | x |  |
| MIB:ZPL:03202 | *Juglans regia* L. | HE963521 | HE966942 | HE966671 |  |  |  |
| MIB:ZPL:03400 | *Juniperus communis* L. | HE963522 | HE966943 | HE966672 |  |  |  |
| MIB:ZPL:03228(i-iii) | *Koeleria lobata* (M. Bieb.) Roem. & Schult. | HE963523  HE963524  HE963525 | - | HE966673  HE966674  HE966675 |  | x |  |
| MIB:ZPL:03984 | *Koelreuteria paniculata* Laxm. | HE963526 | HE967422 | HE966676 |  |  | x |
| MIB:ZPL:03390 | *Laburnum anagyroides* Medik. subsp*. anagyroides* | HE963527 | HE967423 | - |  |  | x |
| MIB:ZPL:03687 | *Lactuca muralis* (L.) Gaertn*.* | HE963528 | HE970719 | HE966677 |  |  |  |
| MIB:ZPL:03561 | *Lactuca serriola* L. | HE963529 | HE967424 | HE966678 |  |  |  |
| MIB:ZPL:03189 | *Lamium maculatum* L. | HE963530 | HE966944 | HE966679 | x |  |  |
| MIB:ZPL:03198 | *Lathyrus latifolius L.* | HE963531 | HE967425 | HE966680 |  |  |  |
| MIB:ZPL:03044 | *Lathyrus niger* (L.) Bernh*.* | HE963532 | HE967426 | HE966681 |  |  |  |
| MIB:ZPL:03226(i-iii) | *Lathyrus pratensis* L. subsp*. pratensis* | HE963533  HE963534  HE963535 | HE967427  HE967428  HE967429 | HE966682  HE966683  HE966684 |  |  |  |
| MIB:ZPL:03036 | *Laurus nobilis* L. | HE963536 | HE966945 | HE966685 |  |  |  |
| MIB:ZPL:03990 | *Lavandula angustifolia* Mill. subsp*. angustifolia* | HE963537 | HE967430 | HE966686 |  |  |  |
| MIB:ZPL:03234 | *Lepidium draba* L. subsp*. draba* | HE963538 | HE967431 | HE966687 |  | x |  |
| MIB:ZPL:03079 | *Ligustrum vulgare* L*.* | HE963539 | HE966946 | HE966688 |  |  |  |
| MIB:ZPL:03235 | *Linum tenuifolium* L*.* | HE963540 | HE966947 | HE966689 |  | x |  |
| MIB:ZPL:03982 | *Liriodendron tulipifera* L. | HE963541 | HE966948 | HE966690 |  |  | x |
| MIB:ZPL:03214 | *Lolium perenne* L. | HE963542 | HE967432 | HE966691 |  | x |  |
| MIB:ZPL:03571(i-iii) | *Lonicera japonica* Thunb*.* | HE963543  HE963544  HE963545 | HE970720  HE970721  HE970722 | HE966692  HE966693  HE966694 |  |  |  |
| MIB:ZPL:03073 | *Lotus corniculatus* L. subsp*. corniculatus* | HE963546 | HE966949 | HE966695 |  |  |  |
| MIB:ZPL:03549 | *Lunaria annua* L. | HE963547 | HE967433 | HE966696 |  | x |  |
| MIB:ZPL:03063 | *Luzula multiflora* (Ehrh.) Lej*.* | HE963548 | HE966950 | HE966697 |  | x |  |
| MIB:ZPL:03405 | *Malus pumila* Mill. WTF? | HE963549 | HE967434 | HE966698 |  |  |  |
| MIB:ZPL:03184 | *Malva sylvestris* L. subsp*. sylvestris* | HE963550 | HE967435 | HE966699 |  | x |  |
| MIB:ZPL:03250(i-iii) | *Medicago falcata* L. subsp*. falcata* | HE963551  HE963552  HE963553 | HE967436  HE967437  HE967438 | HE966700  HE966701  HE966702 |  |  |  |
| MIB:ZPL:03072(i-iii) | *Medicago lupulina* L*.* | HE963554  HE963555  HE963556 | HE966951  HE966952  HE966953 | HE966703  HE966704  HE966705 |  |  |  |
| MIB:ZPL:03193 | *Medicago minima* (L.) L. | HE963557 | HE966954 | HE966706 |  |  |  |
| MIB:ZPL:03404 | *Medicago sativa* L*.* | HE963558 | HE967439 | HE966707 |  |  |  |
| MIB:ZPL:03986 | *Melia azedarach* L. | HE963559 | HE967440 | HE966708 |  |  |  |
| MIB:ZPL:03563 | *Melilotus albus* Medik*.* | HE963560 | HE967441 | HE966709 |  |  |  |
| MIB:ZPL:03401 | *Melilotus officinalis* (L.) Lam*.* | HE963561 | HE970723 | HE966710 |  |  |  |
| MIB:ZPL:03040 | *Melittis melissophyllum* L. s.l. | HE963562 | HE966955 | HE966711 | x |  |  |
| MIB:ZPL:03237 | *Mentha longifolia* (L.) Huds*.* | HE963563 | HE967442 | HE966712 | x |  |  |
| MIB:ZPL:03125 | *Mentha pulegium* L. subsp*. pulegium* | HE963564 | HE966956 | HE966713 | x |  |  |
| MIB:ZPL:03677 | *Mercurialis annua* L*.* | HE963565 | HE967443 | HE966714 | x |  |  |
| MIB:ZPL:03124 | *Muscari comosum* (L.) Mill*.* | HE963566 | - | HE966715 |  | x |  |
| MIB:ZPL:03543 | *Myrtus communis* L. subsp*. communis* | HE963567 | HE967444 | HE966716 |  |  |  |
| MIB:ZPL:03675 | *Nerium oleander* L. subsp*. oleander* | HE963568 | HE966957 | HE966717 |  |  |  |
| MIB:ZPL:03562 | *Nymphaea alba* L. | HE963569 | HE967445 | - |  |  |  |
| MIB:ZPL:03402 | *Olea europaea* L. | HE963570 | HE967446 | HE966718 |  |  |  |
| MIB:ZPL:03247 | *Onobrychis arenaria* (Kit.) DC*.* subsp*. tommasinii* (Jord.) Asch. & Graebn. | HE963571 | HE967447 | HE966719 |  |  |  |
| MIB:ZPL:03664 | *Ononis spinosa* L. subsp*. spinosa* | HE963572 | HE967448 | HE966720 |  |  |  |
| MIB:ZPL:03070 | *Orobanche gracilis* Sm. | HE963573 | HE967449 | - |  |  |  |
| MIB:ZPL:03569 | *Orobanche hederae* Duby | HE963574 | HE967450 | - |  |  |  |
| MIB:ZPL:03539 | *Ostrya carpinifolia* Scop*.* | HE963575 | HE967451 | HE966721 |  |  | x |
| MIB:ZPL:03223 | *Oxalis articulata* Savigny | HE963576 | HE967452 | HE966722 |  |  |  |
| MIB:ZPL:03190 | *Oxalis corniculata* L. | HE963577 | HE966958 | HE966723 |  |  |  |
| MIB:ZPL:03386 | *Oxalis stricta* L*.* | HE963578 | HE967453 | - |  |  |  |
| MIB:ZPL:03217 | *Papaver rhoeas* L. subsp*. rhoeas* | HE963579 | HE966959 | HE966724 |  |  |  |
| MIB:ZPL:03134 | *Parietaria officinalis* L*.* | HE963580 | HE966961 | HE966725 |  | x |  |
| MIB:ZPL:03240 | *Parietaria judaica* L*.* | HE963581 | HE966960 | - |  | x |  |
| MIB:ZPL:03135 | *Parthenocissus quinquefolia* (L.) Planch. | HE963582 | HE966962 | HE966726 |  |  |  |
| MIB:ZPL:03554 | *Parthenocissus tricuspidata* (Siebold & Zucc.) Planch. | HE963583 | - | HE966727 |  |  |  |
| MIB:ZPL:03999 | *Persicaria lapathifolia* (L.) Delarbre subsp. *lapathifolia* | HE963584 | HE967454 | HE966728 |  | x |  |
| MIB:ZPL:03231(i-iii) | *Petrorhagia saxifraga* (L.) Link subsp. *saxifraga* | HE963585  HE963586  HE963587 | HE967455  HE967456  HE967457 | HE966729  HE966730  HE966731 | x |  |  |
| MIB:ZPL:03530 | *Phillyrea angustifolia* L*.* | HE963588 | HE967458 | HE966732 |  |  |  |
| MIB:ZPL:04009 | *Phytolacca americana* L. | HE963589 | HE967459 | HE966733 |  |  |  |
| MIB:ZPL:04010 | *Picea abies* (L.) H. Karst*.* | HE963590 | HE966963 | HE966734 |  |  |  |
| MIB:ZPL:03376(i-iii) | *Picris hieracioides* L. subsp*. spinulosa* (Bertol. ex Guss.) Arcang*.* | HE963591  HE963592  HE963593 | HE970724  HE970725  HE970726 | HE966735  HE966736  HE966737 |  | x |  |
| MIB:ZPL:03107 | *Pinus nigra* J.F. Arnold subsp. *nigra* | HE963594 | HE966964 | HE966738 |  |  |  |
| MIB:ZPL:03094 | *Piptatherum miliaceum* (L.) Coss*.* subsp*. miliaceum* | HE963595 | HE966965 | HE966739 |  | x |  |
| MIB:ZPL:03087 | *Pittosporum tobira* (Thunb.) W.T*.* Aiton | HE963596 | HE966966 | HE966740 |  |  |  |
| MIB:ZPL:03114 | *Plantago holosteum* Scop*.* | HE963597 | HE966967 | HE966741 |  | x |  |
| MIB:ZPL:03104 | *Plantago lanceolata* L*.* | HE963598 | HE966968 | HE966742 |  | x |  |
| MIB:ZPL:03127 | *Plantago major* L. subsp. *major* | HE963599 | HE966969 | HE966743 |  | x |  |
| MIB:ZPL:03262 | *Poa annua* L*.* | HE963600 | HE967460 | HE966744 |  | x |  |
| MIB:ZPL:03054 | *Poa bulbosa* L. subsp*. bulbosa* | HE963601 | HE966970 | HE966745 |  | x |  |
| MIB:ZPL:03256 | *Poa trivialis* L. subsp*. sylvicola* (Guss.) H. Lindb*.* | HE963602 | HE966971 | HE966746 |  | x |  |
| MIB:ZPL:03546 | *Polycarpon tetraphyllum* (L.) L. subsp*. tetraphyllum* | HE963603 | HE967461 | HE966747 | x |  |  |
| MIB:ZPL:03263 | *Polygonum arenastrum* Boreau subsp. *arenastrum* | HE963604 | HE967462 | HE966748 |  | x |  |
| MIB:ZPL:03985 | *Polypodium cambricum* L. | HE963605 | HE970727 | HE966749 |  |  |  |
| MIB:ZPL:03547 | *Polypodium interjectum* Shivas | HE963606 | - | HE966750 |  |  |  |
| MIB:ZPL:04007 | *Poncirus trifoliata* (L.) Raf. | HE963607 | HE967463 | HE966751 |  |  |  |
| MIB:ZPL:03257 | *Populus nigra* L. | HE963608 | HE967464 | HE966752 |  |  | x |
| MIB:ZPL:03536 | *Populus tremula* L. | HE963609 | HE967465 | HE966753 |  |  | x |
| MIB:ZPL:03669 | *Portulaca oleracea* L. subsp*. oleracea* | HE963610 | HE967466 | HE966754 | x |  |  |
| MIB:ZPL:04517 | *Potentilla indica* (Andrews) Th. Wolf | HE963611 | HE966972 | HE966755 |  |  |  |
| MIB:ZPL:03532 | *Potentilla reptans* L. | HE963612 | HE967467 | HE966756 |  |  |  |
| MIB:ZPL:03560 | *Prunella vulgaris* L. subsp*. vulgaris* | HE963613 | HE967468 | HE966757 | x |  |  |
| MIB:ZPL:03132 | *Prunus avium* L. subsp*. avium* | HE963614 | HE966973 | HE966758 |  |  |  |
| MIB:ZPL:03398 | *Prunus cerasifera* Ehrh. var*. pissardii* (Carriére) L.H.Bailey | HE963615 | HE966975 | HE966759 |  |  |  |
| MIB:ZPL:03265 | *Prunus cerasifera* Ehrh. | HE963616 | HE966974 | HE966760 |  |  |  |
| MIB:ZPL:03551 | *Prunus domestica* L. subsp*. insititia* (L.) Bonnier & Layens | HE963617 | HE967469 | HE966761 |  |  |  |
| MIB:ZPL:03558 | *Prunus laurocerasus* L. | HE963618 | HE967470 | HE966762 |  |  |  |
| MIB:ZPL:03109 | *Prunus mahaleb* L. s.l. | HE963619 | HE966976 | HE966763 |  |  |  |
| MIB:ZPL:03259 | *Prunus persica* (L.) Batsch | HE963620 | HE966977 | HE966764 |  |  |  |
| MIB:ZPL:03120 | *Prunus spinosa* L. subsp*. spinosa* | HE963621 | HE966978 | HE966765 |  |  |  |
| MIB:ZPL:03662 | Pseudolysimachion barrelieri (H. Schott ex Roem. & Schlt.) Holub | HE963622 | HE967471 | HE966766 | x |  |  |
| MIB:ZPL:03531 | *Punica granatum* L*.* | HE963623 | HE967472 | HE966767 |  |  |  |
| MIB:ZPL:03670 | *Pyracantha coccinea* M. Roem*.* | HE963624 | HE970728 | HE966768 |  |  |  |
| MIB:ZPL:03105 | *Quercus ilex* L. subsp*. ilex* | HE963625 | HE967473 | HE966769 |  |  |  |
| MIB:ZPL:03049 | *Quercus petraea* (Matt.) Liebl. subsp*. petraea* | HE963626 | HE966979 | HE966770 |  |  | x |
| MIB:ZPL:03050 | *Quercus pubescens* Willd. subsp*. pubescens* | HE963627 | HE966980 | HE966771 |  |  | x |
| MIB:ZPL:03074 | *Ranunculus bulbosus* L*.* | HE963628 | HE967474 | HE966772 |  |  |  |
| MIB:ZPL:03106 | *Reseda lutea* L. subsp*. lutea* | HE963629 | HE967475 | HE966773 |  |  |  |
| MIB:ZPL:03681 | *Ribes nigrum* L*.* | HE963630 | HE967476 | HE966774 |  |  |  |
| MIB:ZPL:03046 | *Robinia pseudoacacia* L*.* | HE963631 | HE970729 | HE966775 |  |  | x |
| MIB:ZPL:03219 | *Rosa agrestis* Savi | HE963632 | HE966981 | HE966776 |  |  |  |
| MIB:ZPL:03103 | *Rosa canina* L*.* | HE963633 | HE966982 | HE966777 |  |  |  |
| MIB:ZPL:03196 | *Rosa micrantha* Borrer ex Sm*.* | HE963634 | HE966983 | HE966778 |  |  |  |
| MIB:ZPL:03403 | *Rosmarinus officinalis* L. | HE963635 | HE967477 | HE966779 |  |  |  |
| MIB:ZPL:03399 | *Rubia peregrina* L. subsp*. longifolia* (Poir.) O. Bolòs | HE963636 | HE967478 | HE966780 |  |  |  |
| MIB:ZPL:03266 | *Rubus canescens* DC. | - | HE967479 | HE966781 |  |  |  |
| MIB:ZPL:03221 | *Rubus ulmifolius* Schott | HE963637 | HE967480 | HE966782 |  |  |  |
| MIB:ZPL:03186(i-iii) | *Rumex obtusifolius* L. subsp*. obtusifolius* | HE963638  HE963639  HE963640 | HE966984  HE966985  HE966986 | HE966783  HE966784  HE966785 |  | x |  |
| MIB:ZPL:03249 | *Rumex pulcher* L. subsp*. pulcher* | HE963641 | HE967481 | HE966786 |  | x |  |
| MIB:ZPL:03039 | *Ruscus aculeatus* L*.* | HE963642 | HE966987 | HE966787 |  |  |  |
| MIB:ZPL:03540 | *Ruscus hypoglossum* L*.* | - | - | HE966788 |  | x |  |
| MIB:ZPL:03572 | *Salvia officinalis* L*.* | HE963643 | HE967482 | HE966789 | x |  |  |
| MIB:ZPL:03556 | *Salvia pratensis* L. subsp*. pratensis* | HE963644 | - | HE966790 | x |  |  |
| MIB:ZPL:03091 | *Sambucus nigra* L. | HE963645 | HE967483 | HE966791 |  |  |  |
| MIB:ZPL:03085 | *Sanguisorba minor* Scop. | HE963646 | HE966988 | HE966792 |  |  |  |
| MIB:ZPL:03683(i-iii) | *Santolina chamaecyparissus* L*.* | HE963647  HE963648  HE963649 | HE970730  HE970731  HE970732 | HE966793  HE966794  HE966795 |  |  |  |
| MIB:ZPL:03384 | *Saponaria officinalis* L*.* | HE963650 | HE967484 | - | x |  |  |
| MIB:ZPL:03668 | *Scabiosa triandra* L*.* | HE963651 | HE970733 | HE966796 |  |  |  |
| MIB:ZPL:03393 | *Scirpoides holoschoenus* (L.) Soják | HE963652 | HE967485 | HE966797 |  | x |  |
| MIB:ZPL:03378 | *Scrophularia canina* L. subsp*. canina* | HE963653 | HE967486 | HE966798 |  |  |  |
| MIB:ZPL:03218 | *Securigera varia* (L.) Lassen | HE963654 | HE966989 | HE966799 |  |  |  |
| MIB:ZPL:03242(i-iii) | *Sedum sexangulare* L*.* | HE963655  HE963656  HE963657 | HE966990  HE966991  HE966992 | HE966800  HE966801  HE966802 |  | x |  |
| MIB:ZPL:03673 | *Senecio gibbosus* (Guss.) DC. subsp*. gibbosus* | HE963658 | HE970734 | HE966803 |  |  |  |
| MIB:ZPL:03688(i-iii) | *Senecio inaequidens* DC*.* | HE963659  HE963660  HE963661 | HE970735  HE970736  HE970737 | HE966804  HE966805  HE966806 |  | x |  |
| MIB:ZPL:03227 | *Senecio jacobaea* L*.* | HE963662 | HE970738 | HE966807 |  |  |  |
| MIB:ZPL:03216 | *Senecio vulgaris* L. | HE963663 | HE970739 | HE966808 |  |  |  |
| MIB:ZPL:03100 | *Sesleria autumnalis* (Scop.) F.W*.* Schultz | HE963664 | HE966993 | HE966809 |  | x |  |
| MIB:ZPL:03987 | *Setaria pumila* (Poir.) Roem. & Schult. | HE963665 | HE966994 | HE966810 |  | x |  |
| MIB:ZPL:03077 | *Sherardia arvensis* L*.* | HE963666 | HE966995 | HE966811 |  |  |  |
| MIB:ZPL:03082(i-iii) | *Silene latifolia* Poir. subsp*. alba* (Mill.) Greuter & Burdet | HE963667  HE963668  HE963669 | HE967487  HE967488  HE967489 | HE966812  HE966813  HE966814 | x |  |  |
| MIB:ZPL:03053 | *Silene nutans* L. subsp*. nutans* | HE963670 | HE967490 | HE966815 | x |  |  |
| MIB:ZPL:03092(i-iii) | *Silene vulgaris* (Moench) Garcke subsp. *vulgaris* | HE963671  HE963672  HE963673 | HE966996  HE966997  HE966998 | HE966816  HE966817  HE966818 | x |  |  |
| MIB:ZPL:03528(i-iii) | *Silybum marianum* (L.) Gaertn*.* | HE963674  HE963675  HE963676 | HE970740  HE970741  HE970742 | HE966819  HE966820  HE966821 |  |  |  |
| MIB:ZPL:03204(i-iii) | *Sisymbrium officinale* (L.) Scop*.* | HE963677  HE963678  HE963679 | HE966999  HE967000  HE967001 | HE966822  HE966823  HE966824 |  |  |  |
| MIB:ZPL:03253 | *Solanum dulcamara* L*.* | HE963680 | HE967491 | HE966825 |  |  |  |
| MIB:ZPL:03992 | *Solanum lycopersicum* L. | HE963681 | HE967492 | HE966826 |  |  |  |
| MIB:ZPL:04005 | *Solanum nigrum* L*.* | HE963682 | HE967493 | HE966827 |  | x |  |
| MIB:ZPL:03385 | *Solanum villosum* Mill. subsp*. alatum* (Moench) Edmonds | HE963683 | HE967494 | HE966828 |  | x |  |
| MIB:ZPL:03110(i-iii) | *Sonchus asper* (L.) Hill subsp*. asper* | HE963684  HE963685  HE963686 | HE970743  HE970744  HE970745 | HE966829  HE966830  HE966831 |  |  |  |
| MIB:ZPL:03052 | *Sorbus domestica* L. | HE963687 | HE967002 | - |  |  |  |
| MIB:ZPL:03090 | *Sorbus torminalis* (L.) Crantz | HE963688 | HE967495 | HE966832 |  |  |  |
| MIB:ZPL:03559 | *Sorghum halepense* (L.) Pers*.* | HE963689 | HE967003 | - |  | x |  |
| MIB:ZPL:03098 | *Spartium junceum* L. | HE963690 | HE967004 | HE966833 |  |  | x |
| MIB:ZPL:03379 | *Spiraea chamaedryfolia* L*.* | HE963691 | HE967496 | HE966834 |  |  | x |
| MIB:ZPL:03573 | *Spirodela polyrhiza* (L.) Schleid. | HE963692 | HE967005 | HE966835 |  |  |  |
| MIB:ZPL:03101 | *Stachys officinalis* (L.) Trevis. | HE963693 | HE967006 | HE966836 | x |  |  |
| MIB:ZPL:03131(i-iii) | *Stellaria media* (L.) Vill. subsp*. media* | HE963694  HE963695  HE963696 | HE967007  HE967008  HE967009 | - | x |  |  |
| MIB:ZPL:04510 | *Sternbergia lutea* (L.) Ker Gawl. ex Spreng. | HE963697 | HE967010 | HE966837 |  | x |  |
| MIB:ZPL:03260 | *Syringa vulgaris* L. | HE963698 | HE967497 | HE966838 |  |  | x |
| MIB:ZPL:03205 | *Tamus communis* L. | HE963699 | HE967011 | HE966839 |  | x |  |
| MIB:ZPL:03060 | *Tanacetum corymbosum* (L.) Sch.Bip. subsp*. corymbosum* | HE963700 | - | HE966840 |  |  |  |
| MIB:ZPL:04519 | *Taraxacum laevigatum* (Willd.) DC*.* | HE963701 | HE970746 | HE966841 |  |  |  |
| MIB:ZPL:03126 | *Taraxacum officinale* Weber in Wiggers s.l. | HE963702 | HE970747 | HE966842 |  | x |  |
| MIB:ZPL:03035 | *Taxus baccata* L. | HE963703 | HE967498 | HE966843 |  |  |  |
| MIB:ZPL:03243 | *Teucrium chamaedrys* L. subsp*. chamaedrys* | HE963704 | HE967012 | HE966844 | x |  |  |
| MIB:ZPL:03118 | *Thalictrum minus* L. s.l. | HE963705 | HE967499 | - |  |  |  |
| MIB:ZPL:03211 | *Thesium humifusum* DC. | HE963706 | HE967500 | HE966845 |  | x |  |
| MIB:ZPL:03123 | *Thymus pulegioides* L. | HE963707 | HE967501 | HE966846 | x |  |  |
| MIB:ZPL:03537 | *Tilia cordata* Mill*.* | HE963708 | HE967502 | HE966847 |  |  | x |
| MIB:ZPL:04518 | *Trachycarpus fortunei* (Hook.) H. Wendl. | HE963709 | HE967013 | HE966848 |  |  |  |
| MIB:ZPL:03116 | *Tragopogon dubius* Scop. | HE963710 | HE970748 | HE966849 |  | x |  |
| MIB:ZPL:03692 | *Trifolium arvense* L. subsp. *arvense* | - | - | - |  |  |  |
| MIB:ZPL:03230 | *Trifolium campestre* Schreb*.* | HE963711 | HE970749 | HE966850 |  |  |  |
| MIB:ZPL:03115 | *Trifolium montanum* L. subsp*. montanum* | HE963712 | HE967503 | HE966851 |  |  |  |
| MIB:ZPL:03208 | *Trifolium pratense* L. subsp*. pratense* | HE963713 | HE967504 | HE966852 |  |  |  |
| MIB:ZPL:03232 | *Trifolium repens* L. | HE963714 | HE967014 | HE966853 |  |  |  |
| MIB:ZPL:03057 | *Trifolium rubens* L*.* | HE963715 | HE967505 | HE966854 |  |  |  |
| MIB:ZPL:03565 | *Ulmus laevis* Pall. | HE963716 | HE970750 | - |  |  | x |
| MIB:ZPL:03097 | *Ulmus minor* Mill. subsp*. minor* | HE963717 | HE967015 | HE966855 |  |  | x |
| MIB:ZPL:03197 | *Ulmus* x *hollandica* Mill*.* | HE963718 | HE967016 | HE966856 |  |  | x |
| MIB:ZPL:03088 | *Urtica dioica* L. subsp*. pubescens* (Ledeb.) Domin | HE963719 | HE967017 | HE966857 |  |  |  |
| MIB:ZPL:03119 | *Valerianella dentata* (L.) Pollich | HE963720 | HE967018 | HE966858 | x |  |  |
| MIB:ZPL:03117 | *Verbascum chaixii* Vill. subsp*. chaixii* | HE963721 | HE967506 | HE966859 |  | x |  |
| MIB:ZPL:03550 | *Verbascum phlomoides* L*.* | HE963722 | HE967507 | HE966860 |  | x |  |
| MIB:ZPL:03545 | *Verbena officinalis* L*.* | HE963723 | HE967508 | HE966861 |  |  |  |
| MIB:ZPL:03233 | *Veronica arvensis* L. | HE963724 | HE967019 | HE966862 | x |  |  |
| MIB:ZPL:03121 | *Veronica persica* Poir. | HE963725 | HE967509 | HE966863 | x |  |  |
| MIB:ZPL:03693 | *Viburnum tinus* L*.* subsp*. tinus* | HE963726 | HE970751 | HE966864 |  |  |  |
| MIB:ZPL:03099 | *Vicia tenuifolia* Roth subsp*. tenuifolia* | HE963727 | - | HE966865 |  |  |  |
| MIB:ZPL:03133(i-iii) | *Vinca minor* L. | HE963728  HE963729  HE963730 | HE967020  HE967021  HE967022 | HE966866  HE966867  HE966868 | x |  |  |
| MIB:ZPL:03048 | *Vincetoxicum hirundinaria* Medik*.*s.l*.* | HE963731 | HE967023 | HE966869 |  |  |  |
| MIB:ZPL:03388 | *Viola canina* L. | HE963732 | HE967510 | HE966870 |  | x |  |
| MIB:ZPL:04008 | *Viola odorata* L. | HE963733 | HE967024 | - |  | x |  |
| MIB:ZPL:03993 | *Vitis labrusca* L*.* | HE963734 | HE967511 | HE966871 |  |  |  |
| MIB:ZPL:03391 | *Vitis vinifera* L. subsp*. vinifera* | HE963735 | HE967512 | HE966872 |  |  |  |
| MIB:ZPL:03392 | *Wisteria sinensis* (Sims) Sweet | HE963736 | HE967025 | HE966873 |  |  | x |
| **Total** |  | **337** | **323** | **323** | **37** | **105** | **41** |
