# Supplementary material for: DNA Barcoding as an Effective Tool in Improving a Digital Plant Identification System: A Case Study for the Area of Mt. Valerio, Trieste (NE Italy)
Source: PLoS One. 2012 Sep 10;7(9):e43256. doi: 10.1371/journal.pone.0043256 (PMC3438168; doi:10.1371/journal.pone.0043256)
Supplement: Table S2 — Evaluation of intraspecific genetic variability. For a subset of 50 plant species from the Mt. Valerio the mean values of intraspecific variability and the standard error for the three tested makers are provided. Sampling details can be retrieved from Table S1. (DOCX) [file pone.0043256.s002.docx]

**Table S2.** Evaluation of intraspecific genetic variability. For a subset of 50 plant species from the Mt. Valerio the mean values of intraspecific variability and the standard error for the three tested makers are provided. Sampling details can be retrieved from Table S1.

| **Voucher number** | **Species name** | **mean of K2P intraspecific variability ±SE (%)** | | |
| --- | --- | --- | --- | --- |
|  |  | ***rbcL*** | ***matK*** | ***trnH-psbA*** |
| MIB:ZPL:04000 (i-iii) | *Ambrosia artemisiifolia* L. | 0.0 (0.0) | 0.0 (0.0) | 0.8 (0.5) |
| MIB:ZPL:03553 (i-iii) | *Arctium minus* (Hill) Bernh. | 0.0 (0.0) | 0.0 (0.0) | 0.0 (0.0) |
| MIB:ZPL:03209 (i-iii) | *Arenaria serpyllifolia* L. subsp. *serpyllifolia* | 0.0 (0.0) | 0.0 (0.0) | 0.0 (0.0) |
| MIB:ZPL:03254 (i-iii) | *Aristolochia clematitis* L. | 0.0 (0.0) | 0.0 (0.0) | 0.7 (0.4) |
| MIB:ZPL:03071 (i-iii) | *Aristolochia lutea* Desf. | 0.0 (0.0) | 0.0 (0.0) | 0.0 (0.0) |
| MIB:ZPL:03995 (i-iii) | *Atriplex patula* L. | 0.2 (0.2) | 0.1 (0.1) | 1.6 (1.0) |
| MIB:ZPL:03994 (i-iii) | *Berberis thunbergii* DC. | 0.0 (0.0) | 0.0 (0.0) | 0.0 (0.0) |
| MIB:ZPL:04006 (i-iii) | *Buxus sempervirens* L. | 0.0 (0.0) | 0.5 (0.1) | 0.0 (0.0) |
| MIB:ZPL:03062 (i-iii) | *Calluna vulgaris* (L.) Hull | 0.0 (0.0) | 0.0 (0.0) | 0.0 (0.0) |
| MIB:ZPL:04002 (i-iii) | *Cardamine hirsuta* L. | 0.0 (0.0) | 0.0 (0.0) | 0.4 (0.3) |
| MIB:ZPL:03195 (i-iii) | *Carex flacca* Schreb. subsp*. flacca* | 0.0 (0.0) | 0.0 (0.0) | 0.0 (0.0) |
| MIB:ZPL:03112 (i-iii) | *Carex pairae* F.W. Schultz | 0.0 (0.0) | 0.0 (0.0) | 0.0 (0.0) |
| MIB:ZPL:03983 (i-iii) | *Catalpa bignonioides* Walter | 0.0 (0.0) | 0.0 (0.0) | 0.0 (0.0) |
| MIB:ZPL:03567 (i-iii) | *Catalpa speciosa* (Warder) Engelm*.* | 0.0 (0.0) | 0.0 (0.0) | 0.0 (0.0) |
| MIB:ZPL:03183 (i-iii) | *Chaerophyllum temulum* L. | 0.0 (0.0) | 0.0 (0.0) | 0.0 (0.0) |
| MIB:ZPL:03996 (i-iii) | *Chenopodium ambrosioides* L. | 0.1 (0.1) | 0.2 (0.2) | 0.0 (0.0) |
| MIB:ZPL:03665 (i-iii) | *Cirsium vulgare* (Savi) Ten. subsp. *vulgare* | 0.0 (0.0) | 0.0 (0.0) | 0.6 (0.5) |
| MIB:ZPL:03127 (i-iii) | *Cistus salviifolius* L*.* | 0.0 (0.0) | 0.0 (0.0) | - |
| MIB:ZPL:03130 (i-iii) | *Cornus sanguinea* L. subsp*. hungarica* (Kárpáti) Soó | 0.0 (0.0) | 0.0 (0.0) | 1.0 (0.1) |
| MIB:ZPL:03381 (i-iii) | *Crepis neglecta* L. | 0.0 (0.0) | 0.5 (0.3) | 1.5 (0.6) |
| MIB:ZPL:03394 (i-iii) | *Crepis setosa* Haller f. | 0.0 (0.0) | 0.0 (0.0) | 0.0 (0.0) |
| MIB:ZPL:03122 (i-iii) | *Dianthus sylvestris* Wulfen subsp*. tergestinus* (Rchb.) Hayek | 0.0 (0.0) | 0.0 (0.0) | 0.0 (0.0) |
| MIB:ZPL:03380 (i-iii) | *Diplotaxis tenuifolia* (L.) DC*.* | 0.0 (0.0) | 0.0 (0.0) | 1.1 (0.5) |
| MIB:ZPL:03187 (i-iii) | *Erigeron annuus* (L.) Desf. subsp*. annuus* | 0.0 (0.0) | 0.0 (0.0) | 0.0 (0.0) |
| MIB:ZPL:03236 (i-iii) | *Erigeron sumatrensis* Retz. | 0.0 (0.0) | 0.0 (0.0) | 0.0 (0.0) |
| MIB:ZPL:03529 (i-iii) | *Euphorbia characias* L. subsp*. wulfenii* (Hoppe ex Koch) A.R.Sm*.* | 0.0 (0.0) | 0.0 (0.0) | 0.0 (0.0) |
| MIB:ZPL:03056 (i-iii) | *Euphorbia cyparissias* L. | 0.0 (0.0) | 0.4 (0.2) | 0.6 (0.5) |
| MIB:ZPL:03210 (i-iii) | *Euphorbia peplus* L. | 0.3 (0.2) | 0.4 (0.3) | 0.0 (0.0) |
| MIB:ZPL:03383 (i-iii) | *Galinsoga quadriradiata* Ruiz & Pav*.* | 0.0 (0.0) | 0.0 (0.0) | 0.0 (0.0) |
| MIB:ZPL:03037 (i-iii) | *Hedera helix* L. s.l. | 0.0 (0.0) | 0.2 (0.2) | 0.0 (0.0) |
| MIB:ZPL:04508 (i-iii) | *Hieracium racemosum* Waldst. & Kit. ex Willd. | - | 0.4 (0.3) | - |
| MIB:ZPL:03067 (i-iii) | *Inula hirta* L. | 0.7 (0.3) | 0.2 (0.1) | 0.2 (0.0) |
| MIB:ZPL:03228 (i-iii) | *Koeleria lobata* (M. Bieb.) Roem. & Schult. | 0.0 (0.0) | - | 0.0 (0.0) |
| MIB:ZPL:03226 (i-iii) | *Lathyrus pratensis* L. subsp*. pratensis* | 0.0 (0.0) | 0.0 (0.0) | 1.4 (0.6) |
| MIB:ZPL:03571 (i-iii) | *Lonicera japonica* Thunb*.* | 0.0 (0.0) | 0.0 (0.0) | 1.7 (0.5) |
| MIB:ZPL:03250 (i-iii) | *Medicago falcata* L. subsp*. falcata* | 0.0 (0.0) | 0.0 (0.0) | 0.0 (0.0) |
| MIB:ZPL:03072 (i-iii) | *Medicago lupulina* L*.* | 0.0 (0.0) | 0.0 (0.0) | 0.6 (0.3) |
| MIB:ZPL:03231 (i-iii) | *Petrorhagia saxifraga* (L.) Link subsp. *saxifraga* | 0.0 (0.0) | 0.0 (0.0) | 0.0 (0.0) |
| MIB:ZPL:03376 (i-iii) | *Picris hieracioides* L. subsp*. spinulosa* (Bertol. ex Guss.) Arcang*.* | 0.0 (0.0) | 0.0 (0.0) | 0.0 (0.0) |
| MIB:ZPL:03186 (i-iii) | *Rumex obtusifolius* L. subsp*. obtusifolius* | 0.0 (0.0) | 0.0 (0.0) | 0.0 (0.0) |
| MIB:ZPL:03683 (i-iii) | *Santolina chamaecyparissus* L*.* | 0.0 (0.0) | 0.0 (0.0) | 0.0 (0.0) |
| MIB:ZPL:03242 (i-iii) | *Sedum sexangulare* L*.* | 0.0 (0.0) | 0.0 (0.0) | 0.0 (0.0) |
| MIB:ZPL:03688 (i-iii) | *Senecio inaequidens* DC*.* | 0.0 (0.0) | 0.7 (0.1) | 0.0 (0.0) |
| MIB:ZPL:03082 (i-iii) | *Silene latifolia* Poir. subsp*. alba* (Mill.) Greuter & Burdet | 0.0 (0.0) | 0.5 (0.2) | 1.8 (0.8) |
| MIB:ZPL:03092 (i-iii) | *Silene vulgaris* (Moench) Garcke subsp. *vulgaris* | 0.3 (0.3) | 0.0 (0.0) | 1.6 (0.9) |
| MIB:ZPL:03528 (i-iii) | *Silybum marianum* (L.) Gaertn*.* | 0.0 (0.0) | 0.3 (0.3) | 0.0 (0.0) |
| MIB:ZPL:03204 (i-iii) | *Sisymbrium officinale* (L.) Scop*.* | 0.0 (0.0) | 0.0 (0.0) | 0.0 (0.0) |
| MIB:ZPL:03110 (i-iii) | *Sonchus asper* (L.) Hill subsp*. asper* | 0.0 (0.0) | 0.0 (0.0) | 0.6 (0.6) |
| MIB:ZPL:03131 (i-iii) | *Stellaria media* (L.) Vill. subsp*. media* | 0.0 (0.0) | 0.0 (0.0) | - |
| MIB:ZPL:03133 (i-iii) | *Vinca minor* L. | 0.1 (0.1) | 0.0 (0.0) | 1.9 (1.6) |
